# Evaluating an e-learning program to strengthen the capacity of humanitarian workers in the MENA region: the Humanitarian Leadership Diploma

**DOI:** 10.1186/s13031-022-00460-2

**Published:** 2022-05-20

**Authors:** Shadi Saleh, Dayana Brome, Rania Mansour, Tracy Daou, Amar Chamas, Hady Naal

**Affiliations:** 1 Global Health Institute, American University of Beirut, Beirut, Lebanon; 2 Faculty of Health Sciences, American University of Beirut, Beirut, Lebanon; 3 St George’s Hospital Medical School, St George’s University of London, London, UK

**Keywords:** Middle East and North Africa, E-learning, Online learning, Humanitarian crisis, Conflict, Global health, Humanitarian workers

## Abstract

**Background:**

The Middle East and North Africa (MENA) region is consistently plagued with humanitarian crises while having little response capacity. Despite their obvious growing need, there exist limited educational opportunities for humanitarian workers to develop their capacity in humanitarian topics. The present study evaluates an online training program, the Humanitarian Leadership Diploma (HLD), which targeted humanitarian workers across the MENA region.

**Methods:**

A mixed-methods design was used, comprising short and long-term quantitative and qualitative data, targeting individual and organizational-level outcomes. A total of 28 humanitarian workers across the MENA region enrolled in the program starting September 2019 until October 2020, 18 of which completed the full diploma. Short-term quantitative data such as knowledge assessments, course evaluations, and reflective commentaries were collected from all learners, whereas long-term qualitative data was collected only from those who completed the full diploma and from peers at their organizations, 6 months after completion. Data was triangulated, analyzed using qualitative content analysis, and reported as themes.

**Results:**

The program was overall successful given multiple factors reported by participants such as enhanced knowledge, high satisfaction, and improved practice, with some important challenges being identified. Themes under the strengths category related to (1) online learning, (2) significance of diploma, (3) course content, (4) instructors, (5) transfer of learning into practice, and (6) personal development. Themes under the challenges category related to (1) barriers to applying changes in behavior and performance, (2) engagement and interaction, and (3) pedagogical approach.

**Conclusion:**

This is one of very few evaluations of locally developed and delivered online learning programs for humanitarian actors in the MENA region. The findings are especially important as they may inform researchers and humanitarian actors looking to design and deliver similar programs in the MENA region or other fragile settings. Key recommendations are discussed in the manuscript, and include to combine synchronous and asynchronous approaches, design concise course materials, limit theoretical pedagogical approaches, ensure topics are contextualized to the region, and consider continuous engagement strategies for learners.

## Background

Armed conflicts and civil wars in the Middle East and North Africa (MENA) region have been ongoing since World War II and have been exacerbated since the 2011 Arab Spring. As of 2021, regional conflicts include the Israel-Palestine War, Kurdistan-Turkey Conflict, Yemen Crisis, Syria Crisis, Egypt Subnational Conflict, Libyan Civil War, and the Iraqi Insurgency [1]. The impact of these conflicts on surrounding MENA countries is equally significant; for example, Lebanon is currently hosting the largest number of refugees per capita [2] and Turkey is hosting the largest number of refugees worldwide [3]. According to the State of the Humanitarian System (SOHS) 2018 Summary Report by the Active Learning Network for Accountability and Performance (ALNAP) of the Overseas Development Institute, in 2017, 23% of people in need of humanitarian assistance and protection, which may encompass food, shelter, clothing, medical supplies, legal support, protection, and infrastructure, were living in just three countries and all within the MENA region: Yemen, Syria, and Turkey [4]. By definition, humanitarian crises present critical threats to the health, safety, security, or wellbeing of a community. Most significantly are the health consequences of armed conflict on civilians which have been extensively documented as being due to multiple factors including the displacement of populations, the heightened risk of disease transmission, the demanding health needs of populations living in chronic fragility and unstable socio-political circumstances, the increasing attacks against healthcare institutions, brain drain of knowledgeable and skilled health professionals, and the breakdown of fragile health and social services [5–9]. A recent study in the Lancet has quantified the impact of armed conflict on vulnerable populations: among women of reproductive age, those living near conflict have three times higher mortality than those in peaceful settings; additionally, conflicts globally between 1995 and 2015 account for approximately 10 million deaths in children younger than 5 years of age [10].

Such circumstances have naturally honed the presence of humanitarian organizations whose position as non-state actors allows them to play a crucial role by providing assistance via immediate emergency medical aid, research, infrastructure, and economic development, as well as social and cultural services and goods [11, 12]. As an example, Lebanon is now the United Nations’ High Commission for Refugees (UNHCR) largest single-country operation, where it works closely with the Government and other partners to provide protection and assistance to refugees, stateless persons, and affected Lebanese communities [2, 13]. Over the past two decades, the presence of humanitarian organizations in the MENA region has increased; according to the UN Department of Economic and Social Affairs NGO Branch, there are 831 NGOs operating across MENA countries and cooperating with the Economic and Social Committee (ECOSOC) of the United Nations as of June 2021 [14]. In comparison, there were only 162 NGOs operating with such status in the MENA region in 2009 [15, 16]. These numbers do not reflect the true or total number of humanitarian organizations working in the region as it does not account for those operating without recognition by the UN Economic and Social Committee. Thus, the number of organizations working on all sectors of humanitarian assistance in the region is expected to be much greater.

Nevertheless, the rapid growth of organizations in the region has been coupled by the disproportionate development and expansion of human capacity throughout the humanitarian sector. As such, despite an approximate 20% increase in funding for humanitarian organizations over the time period of 2015–2018, the humanitarian system still does not have sufficient human and material resources to cover the needs of people affected by humanitarian crises [4]. This may partially be because many services provided by humanitarian organizations induce dependency by the populations they serve, resulting in a need for such organizations to continuously provide assistance rather than prioritize growth, development, and efficiency. A well-established key factor to enhance the efficiency of humanitarian aid operations is the presence of quality leadership and qualified personnel who possess the relevant technical capacity and contextualized knowledge to work in the setting of interest [17, 18]. Effective leadership has been stated to involve the appropriate motivation, management, inspiration, and remuneration of employees in addition to the presence of analytical skills necessary to promote creativity and innovation within an organization [19, 20]. Effective leadership is also likely to foster a culture of independence and accountability for the situation or state of an organization, as opposed to relying on external actors for support*. Research has proven that leadership that can accomplish the aforementioned is more likely to positively affect organizational performance, as has been measured via reduced staff turnover rates, better employee performance evaluations, increased quality and reduced cost of organizational outcomes, and improved financial performance of the organization [19, 21]. In a 2010 pilot study for The State of the Humanitarian System (SOHS), humanitarian workers indicated the lack of strong leadership and coordination as being both the most fundamental issue and the most difficult challenge to overcome within humanitarian [22]. Other field reports indicated problems revolving around high staff turnover, the necessity to invest more in human resource management systems, and the repeated concern of insufficient investment in local and national capacities [23–25]. A subsequent study by ALNAP attempted to identify qualities essential for effective leadership and the organization later described initiatives to enhance the leadership skills of humanitarian workers [26]. Still, in the SOHS 2018 Report, less than half of humanitarian workers considered leadership as good or excellent [4]. The report also highlighted that performance was particularly poor in conflict settings, demonstrating the enduring struggle to effectively build the leadership capacity of humanitarian workers in fragile settings.

Humanitarian workers across fragile settings, and particularly throughout the MENA region, can be considered to face three main challenges in developing sufficient humanitarian leadership and technical skills. First, it has been argued that the predominant role of international organizations in coordinating and providing humanitarian assistance has the undesirable effect of transferring highly productive local workers to less productive positions, possibly leading to brain drain given their desire to occupy more senior positions [27]. Second, there is limited availability and access to learning opportunities due to the scarcity of context-specific and specialized resources on humanitarian topics, and more importantly the difficulty in conducting in-person training due to security threats and bureaucratic restrictions [28]. This especially came to light during the COVID-19 pandemic, a period where lockdowns and movement restrictions were imposed, and where individuals avoided social gatherings fearing infections. Finally, the complexity of emerging humanitarian emergencies coupled with the numerous and diverse humanitarian initiatives run by humanitarian actors necessitates the implementation of capacity building approaches aimed at developing humanitarian leadership and technical skills that can be applied in diverse environments and across numerous disciplines [29]; as opposed to building the skills of local human resources in silos or per humanitarian sector or organization. It thus becomes essential to develop and deliver engaging, relevant, and accessible training programs on pertinent humanitarian topics at a regional level, that are specifically aimed at building humanitarian leadership and technical capacities and that go beyond formal education in order to ensure wider reach and impact to said actors.

Due to the emerging and complex humanitarian challenges faced in the MENA, the Global Health Institute (GHI) at the American University of Beirut developed the Humanitarian Leadership Diploma (HLD), an online capacity building program under its Academy Division. Given the importance of strong local and regional leadership for humanitarian operations, HLD aims to equip humanitarian workers in the MENA region with relevant and contextualized humanitarian leadership and technical skills to better manage humanitarian projects and resources. HLD adopts innovative e-learning modalities through a hybrid of synchronous and asynchronous delivery methods and consists of eight online courses delivered through GHIs online learning platform, the Global Health Learning and Development (GHLAD) platform. HLD specifically capitalizes on two resources in the region. First, GHIs location in the MENA region provides it access to regional experts in the field of humanitarian leadership who can contribute to and build this diploma based on their work in the field. Second, the availability of technology has enabled distance learning and the virtual classroom to be effective modes of skill-building in low-resourced settings due to its affordability, adaptability, and versatility within humanitarian health contexts, efficiency in allowing information exchange, and opportunity to engage with peers and mentors [30–33]. The first cohort of learners successfully completed the Diploma in October 2020. The present study evaluates the long and short-term effectiveness and impact of the first implementation of this program.

## Methods

### Program development

In August 2017, several meetings were held between GHI and the Humanitarian Leadership Academy (HLA), a partner organization, to agree on the development phase of the diploma. The first phase of development required identification of (a) the competencies of the diploma and (b) the needs of humanitarian workers in the field. During the first stage, a rapid review of academic publications and gray literature was conducted, and the data was analyzed. During the second stage, informal discussions were conducted with representatives from the target population to explore the capacity building needs as well as challenges experienced by regional humanitarian workers. The third stage consisted of triangulating the findings of the review and the informal discussions and synthesizing recommendations to inform the competencies to be covered by the diploma.

**Fig. 1:**
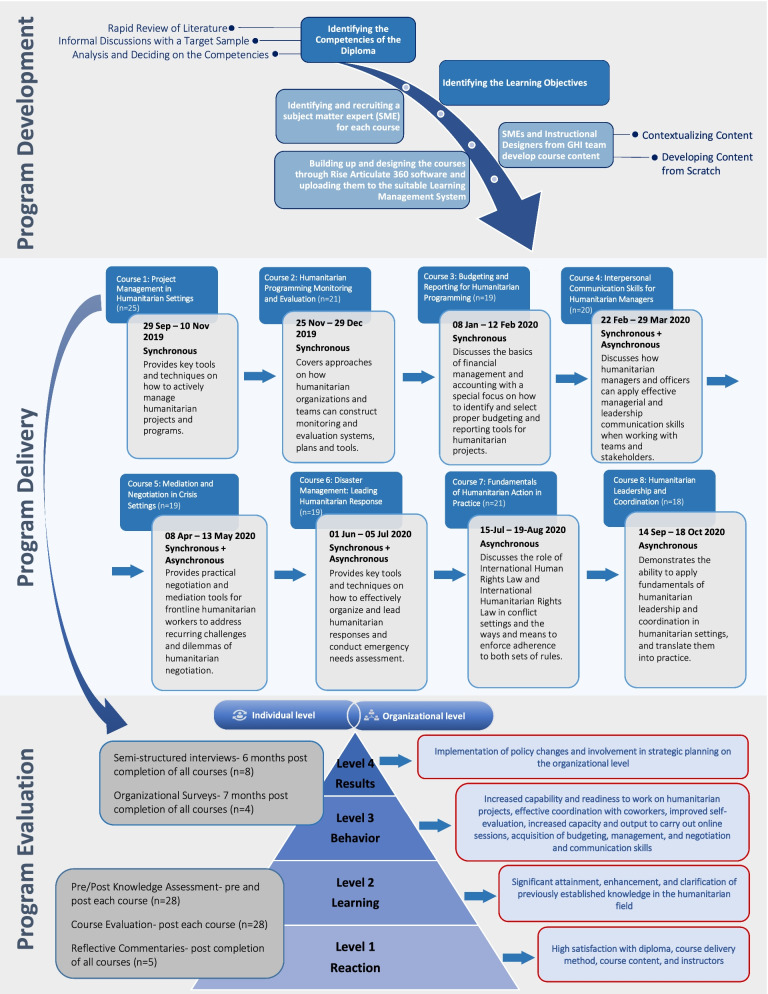
Development, delivery, and evaluation of HLD program

Phase two of development involved identifying learning objectives and recruiting multiple Subject Matter Experts (SME) for each course. SMEs, along with instructional designers from the GHI team, developed the course content, knowledge assessment tools, and cases, and subsequently contextualized and tailored the material to be specifically applicable in the MENA region for discussions. The developed content was cross-validated by content-experts where they further enriched the courses by including personal experiences in the field (see Fig. 1). Following course development, the GHI team created and designed the courses through Rise Articulate 360 software [34]. A total of eight courses were developed and designed (see Table 1). The lectures were then exported as SCORM 1.2 packages and uploaded to the hosting platform: Learning Management System (LMS) Moodle [35]. Each module was then hosted on the online learning platform GHLAD. Interested learners registered through GHLAD and enrolled in the course of interest. They were then immediately redirected to the Moodle dashboard where the courses are hosted.

**Table 1:** Overview of courses

**Course 1:** Project Management in Humanitarian Settings	**Course 2:** Humanitarian Programming Monitoring and Evaluation	**Course 3:** Budgeting and Reporting for Humanitarian Programming	**Course 4:** Interpersonal Communication Skills for Humanitarian Managers
This course provides key tools and techniques on how to actively manage humanitarian projects and programs. It was delivered synchronously and spanned between 29 September and 10 November 2019. A total of 25 registrants were enrolled in this course, 21 completed it, and 4 dropped out	This course covers approaches on how humanitarian organizations and teams can construct monitoring and evaluation systems, plans and tools. It was delivered synchronously and spanned between 25 November and 29 December 2019. A total of 21 registrants were enrolled in this course and completed it	This course discusses the basics of financial management and accounting with a special focus on how to identify and select proper budgeting and reporting tools for humanitarian projects. It was delivered synchronously and spanned between 08 January and 12 February 2020. A total of 19 registrants were enrolled in this course and completed it	This course discusses how humanitarian managers and officers can apply effective managerial and leadership communication skills when working with teams and stakeholders in conflict sensitive humanitarian contexts. It was delivered synchronously and asynchronously, and spanned between 22 February and 29 March, 2020. A total of 20 registrants were enrolled in this course, 19 completed it, and 1 dropped out
**Course 5:** Mediation and Negotiation in Crisis Settings	**Course 6:** Disaster Management: Leading Humanitarian Response	**Course 7:** Fundamentals of Humanitarian Action in Practice	**Course 8:** Humanitarian Leadership and Coordination
This course provides practical negotiation and mediation tools for frontline humanitarian workers to address recurring challenges and dilemmas of humanitarian negotiation in complex environments and humanitarian crisis settings. It was delivered synchronously and asynchronously and spanned between 08 April and 13 May 2020. A total of 19 registrants were enrolled and completed this course	This course provides key tools and techniques on how to effectively organize and lead humanitarian responses and conduct emergency needs assessment in the context of humanitarian disasters. It was delivered synchronously and asynchronously and spanned between 01 June and 05 July 2020. A total of 20 registrants were enrolled in this course, 19 completed it, and 1 dropped out	The course discusses the role of International Human Rights Law (IHL) and International Humanitarian Rights Law (IHRL) in conflict settings and the ways and means to enforce adherence to both sets of rules. It was delivered asynchronously and spanned between 15 July and 19 August 2020. A total of 21 registrants were enrolled in this course, 20 completed it, and 1 dropped out	This course demonstrates the ability to apply fundamentals of humanitarian leadership and coordination in humanitarian settings and translate them into practice according to specific features of the context. It was delivered asynchronously and spanned between 14 September and 18 October 2020. A total of 18 registrants were enrolled in this course and completed it

### Course overview

The eight courses were dissected into easily comprehensible and single-topic sessions. A variety of pedagogical approaches including short presentations, interactive lectures, online discussions, reflective exercises, case studies, assignments, quizzes, and contextualized practical examples and practices from the region were implemented during the sessions. Each course expanded over five weeks for a total of thirty hours. All eight courses were delivered in English and either synchronously, asynchronously, or through a mix of both, all of which were conducted via the online platform GHLAD. Table 1 presents an overview of all courses.

### Recruitment of learners

Learners were recruited through a circulating call for applications on social media platforms. A total of 204 applications were received and reviewed based on the following eligibility criteria: (1) working in humanitarian fields as a mid-to-senior level manager, (2) willingness to commit to the GHI online courses, (3) working in the MENA region, and (4) proficiency in English language. Only 30 individuals were eligible to enroll in the diploma following the exclusion of participants who did not meet the above criteria. Of those eligible, ten were given scholarships to complete the entire diploma. Scholarship recipients were selected based on their curricula vitae and motivation letters, including their years of experience, country of origin (to ensure diversity), and the extent to which they believed the HLD could benefit their work. Eligibility criteria was only applicable to learners who applied for a scholarship. The remaining learners were selected on a first-come first-served basis, with no specific eligibility criteria other than mandatory proficiency in English language and having to pay for the courses they enrolled in. Those who received scholarships were required to maintain a minimum score of 80% on each course, while the remaining learners had to maintain a score of 60%.

A total of 28 learners decided to complete at least one HLD course, 18 of which opted to complete the full diploma of 8 courses. Only those who opted to complete the full diploma were eligible to participate in long-term research activities, however course-related data such as knowledge assessment and course evaluations were reported for all 28 learners. In total 8 learners dropped out from the training program, one of which was a scholarship recipient. Dropouts were determined based on cutting contact midway through the course. While some individuals did not provide justification for dropping out, others mentioned that time constraints and having to prioritize other urgent matters were the reason for discontinuation.

### Design, tools, and procedures

A mixed-methods approach was used to collect short-term and long-term individual as well as organizational-level data through qualitative and quantitative methods. This approach was informed by the Kirkpatrick Model for evaluating training programs through four levels including: (1) reaction, (2) learning, (3) behavior, and (4) results [36]. In order to eliminate potential biases, the researchers who collected the data and carried out the evaluation interviews were separate from the staff who coordinated or who delivered the training. Only the subject matter experts were in charge of developing the course content and delivering the training, none of which are authors of this study.

The research team at GHI contacted learners, supervisors, colleagues, and assistants (who had provided consent to be contacted) through email correspondence to invite them to participate in the study. All learners completed the mandatory pre and post knowledge assessment tests along with the course evaluations as a prerequisite to receiving their course certificate. Learners were also presented with the option to complete a short 500-word narrative summary describing their experiences with the course. Finally, participants who completed the HLD diploma were invited for a semi-structured interview 6 months after completing the diploma, and they were asked to nominate one colleague or supervisor from their organization to complete a survey regarding their performance at work. These organizational surveys were collected 7 months after completing the diploma through the contact information that was provided by learners who participated in the semi-structured interviews.

All participants provided written consent before taking part in research activities, and the study was approved by the Institutional Review Board (IRB) at the American University of Beirut (AUB).

#### Knowledge assessment

To measure changes in learners’ acquired knowledge, knowledge assessment tests were administered to participants before and after the training. These tests were completed online through Moodle [35]. For most courses, questions were presented in the form of multiple-choice questions, while some included True/False and matching questions. Few tests included questions that required learners to arrange steps in a certain order. Each test included 10–20 questions in total and required 7–10 min to complete.

#### Course evaluation

Upon completion of each course, a course evaluation survey was administered to each participant online. This survey aimed to measure participants’ satisfaction with the courses through 16 quantitative questions presented on a 5-point Likert scale (1 = strongly disagree, 5 = strongly agree) addressing the instructor, the course delivery method, and the course content. Four additional open-ended questions were included and asked participants about their opinions on the course, their course expectations, and recommendations to improve the training.

#### Reflective commentaries

An optional reflective commentary tool was administered to participants, asking them to describe through a 500-word narrative essay their experience with the training. Learners were asked to include a story/example/situation describing their experience and to highlight the impact of this training on their skills, attitudes, and practices towards humanitarian leadership. The commentary was delivered to participants online through Lime Survey platform [37] within two weeks following the completion of the diploma. Some of the testimonies collected through this tool are presented in the appendix as vignettes.

#### Semi-structured interviews

Long-term data on the training’s impact on participants was collected through conducting semi-structured interviews (Table 2). Participants who completed a minimum of 6 courses or the full diploma were interviewed 6 months post completion of the program. The interviews aimed to assess participants’ perspective on their experience with the training, their knowledge and practices in humanitarian leadership, and their experiences in initiating or implementing any changes in the practices, policies, and/or strategic plans within their organization. Interview invitation emails were sent out to a total of 18 participants. Only 8 learners however agreed to partake in the interview. That said, data saturation was reached from these initial interviews, and it was agreed by the team that no further interviews are needed. The interviews were conducted in April 2021, included 9 primary talking points followed by prompts and sub questions. The interviews lasted between 20 and 60 min, and were administered in English, Arabic, or a mixture of both depending on the preferences of the interviewee. All interviews were conducted through zoom, recorded following participants’ consent, transcribed verbatim in the original language, and then later translated to English.

**Table 2:** Components of the individual level semi-structured interview guide and organizational level open-ended questions

Tool	Questions
Individual level, semi-structured interview guide	1. Describe your learning experiences during your participation in the training
	2. How did the online learning modality influence your learning process?
	3. Describe your knowledge in humanitarian leadership after your participation in the training
	4. Describe your practices in humanitarian leadership after your participation in the training
	5. How did the training impact your capability to learn new skills?
	6. What are the strengths and weaknesses of the training program?
	7. Describe, if any, changes that you have noticed in your performance within the organization after completing the training
	8. Since your completion of the training have you initiated any changes in the practices and/or policy of your organization?
	9. Based on the knowledge acquired from the courses, have you been able to develop or implement new strategic plans in humanitarian settings?
Organizational level, open-ended questions	1. Describe the performance of the learner within the organization since their completion of the training
	2. Describe the strengths/weaknesses of the [blended, face-to-face, or online] training in facilitating the access of the learner to related education
	3. Describe (if any) barriers that limited the application of the learners’ acquired skills into the organization following their participation in the training

#### Organizational-level surveys

To assess the long-term impact of learners’ participation in the training, and specifically the transfer of knowledge to their organizations, surveys were sent to participants’ line managers or colleagues who were able to comment on the learners’ performance within the organization. The survey included five quantitative questions rated on a 5-point Likert scale (1 = not at all, 5 = very much) followed by three open-ended questions. A total of three participants, occupying the roles of senior project officer, supervisor, and logistic assistant, completed the organizational survey. Seeing the small sample size, we only reported data collected from the open-ended questions.

### Participants and sampling

A total of 28 participants were enrolled in the diploma and completed at least one course. Table 3 delineates participant demographics per course and for those who completed the overall diploma. Most learners were females (56%). The majority were between the ages of 20 and 35 (77.8%), with a mean age of 30.62 (SD = 6.19). More than half of all learners held a Bachelor’s degree (59%), a smaller percentage held a Master’s degree (37%), and only 4% held a Doctoral degree (all of which were medical doctors).

**Table 3:** Demographics of sample across all courses

Demographics	Categories	Course or more n (%)
Gender	Female	15 (56%)
	Male	13 (44%)
Age	20–25	6 (22%)
	26–30	10 (37%)
	31–35	5 (19%)
	36–40	3 (11%)
	41–45	3 (11%)
Educational level	Bachelor’s degree	16 (59%)
	Master’s degree	10 (37%)
	Professional degree	1 (4%)
Nationality	Egyptian	2 (7%)
	Iraqi	1 (4%)
	Jordanian	1 (4%)
	Lebanese	15 (51%)
	Palestinian	1 (4%)
	Sudanese	2 (7%)
	Syrian	2 (7%)
	Turkish	1 (4%)
	Yemenis	1 (4%)
	Tunisian	1 (4%)
	French	1 (4%)
Country of residence	Egypt	2 (7%)
	Iraq	1 (4%)
	Jordan	1 (4%)
	Lebanon	16 (58%)
	Palestine	0 (0)
	Sudan	2 (7%)
	Syria	1 (4%)
	Turkey	1 (4%)
	Yemen	1 (4%)
	Tunisia	1 (4%)
	France	1 (4%)

There was varied representation from national and international organizations, with most learners reporting working in health, livelihoods, food security, protection, education, shelter, and water sanitation and hygiene (WASH) sectors among others. With regards to occupation, participants held officer positions, managerial positions, coordinator positions, and field-related positions. Examples of learners’ job positions included deputy health coordinator, senior health and nutrition officer, food security and livelihood field officer, and shelter and cash project manager among others.

Approximately half of all learners were Lebanese (51%), two were Syrian (7%), two were Sudanese (7%), two were Egyptian (7%); the remaining participants (28%) held nationalities from other countries such as Iraq, Jordan, Palestine, Yemen, Turkey, Tunisia, and France. Most learners resided in Lebanon (59%).

### Data analysis

The quantitative data, including the knowledge assessments and course evaluations, was analyzed using the Statistical Package for the Social Science (SPSS). T-tests were conducted to compare the mean scores on the knowledge assessment tests, while results of the course evaluations were tabulated and presented as frequencies and percentages. Analysis of qualitative data was completed via an inductive approach to content analysis [38, 39]. Data from the interviews was transcribed verbatim into its original language by the research team and then translated to English. To protect the learners’ anonymity, their names were presented as codes. Coding and further analysis of the interviews was performed in parallel independently by two research assistants. Preliminary codes were generated after completing the open coding phase. Codes were then grouped into broader groups based on similarities, and themes were generated based on emerging patterns. The authors met regularly to discuss, analyze, and review the coded responses.

## Results

The results of the short-term and long-term data are presented according to the Kirkpatrick model (Fig. 1). The short-term outcomes included participants’ reactions and learning and were measured through pre and post knowledge assessments, course evaluations administered following each course, and reflective commentaries administered following completion of all eight courses. The long term-outcomes, which include behavioral changes and results at the organizational level, were measured through semi-structured interviews and organizational surveys, conducted 6-months and 7-months post completion of all courses, respectively. The data obtained from semi-structured interviews and organizational surveys was triangulated and merged in the analysis.

### Short term outcomes

#### Knowledge assessment

A significant increase in the test scores from pre to post training was noted in all courses. A statistically significant increase in the test scores from pre to post training was noted for all courses except Course 2, although post training scores were still higher than the pre training scores for Course 2. Table 4 outlines learners’ pre and post test scores for each course.

**Table 4:** Learners’ pre and post-test knowledge assessment stratified by course

Courses	Pre-test		Post-test		Paired t test		
	M	SD	M	SD	*t*	*df*	Sig
Course 1 (n = 25)	59.46	22.16	77.60	11.49	− 2.714	14	**.017**
Course 2 (n = 21)	78.85	9.76	82.18	8.24	− 1.767	17	.095
Course 3 (n = 19)	51.12	4.30	79.34	3.13	− 5.621	18	**.000**
Course 4 (n = 20)	40.07	15.83	65.46	18.27	4.447	18	**.000**
Course 5 (n = 19)	52.63	15.12	77.63	12.28	− 6.673	18	**.000**
Course 6 (n = 19)	57.04	2.74	82.77	3.08	− 5.463	19	**.000**
Course 7 (n = 21)	49.52	3.53	87.34	2.96	− 8.101	19	**.000**
Course 8 (n = 18)	69.41	8.80	84.49	11.52	− 5.409	17	**.000**

Bolder numbers in the table point towards statistical results that are significant (all numbers that are < 0.05)

Course 1 = Project Management in humanitarian settings; course 2 = Humanitarian Programming Monitoring and Evaluation; course 3 = Budgeting and Reporting for Humanitarian Programming; course 4 = Interpersonal Communication Skills for Humanitarian Managers; Course 5 = Mediation and Negotiation in Crisis Settings; Course 6 = Disaster Management: Leading Humanitarian Response; Course 7 = Fundamentals of Humanitarian Action in Practice; Course 8 = Humanitarian Leadership and Coordination

#### Course evaluation survey

Results from course evaluations obtained from learners at the end of each course indicate that the training program was well received, and that satisfaction was high.

Regarding the method of course delivery, most learners agreed or strongly agreed that the course objectives were fulfilled (80.3%) and were clearly stated and presented (83.2%). Most learners also agreed or strongly agreed that the time allocated for the course (77.1%) as well as the pace of the course (77.2%) was appropriate. Additionally, most learners reported that the content was relevant to their needs (74.5%) and that they would take another course through this delivery method (87.3%).

Evaluations of course content demonstrated that the majority of learners agreed or strongly agreed that the course contents were organized and easy to follow (79.8%) and that they obtained useful competences (79.9%). Similarly, a high percentage of learners agreed or strongly agreed that their knowledge and competence in the subject matter increased (86.3%). Most learners reported being satisfied with the overall quality of the courses (78.1%) and would recommend it to others (79.8%). Additionally, many learners agreed or strongly agreed that they would participate in future courses by GHI (91.3%).

Feedback regarding instructors’ performance indicated that most learners agreed or strongly agreed that the instructor was well-prepared and knowledgeable about the topic (86.6%). Most learners also agreed or strongly agreed that the instructor was able to communicate the course’s information clearly and effectively (82.5%) and was able to stimulate students’ interest in the subject (77.1%). Moreover, most learners were satisfied with the overall performance of instructors (83.3%).

Table 5 reports the percentage of participants in agreement (i.e., Agree and Strongly Agree) with each component of the course evaluation; data is also stratified by learning modality.

**Table 5:** Course evaluation results stratified by learning modality and by course or more

Evaluation component	Learners’ evaluation			
	%Synchronous (n = 25)	%Synchronous and asynchronous (n = 20)	%Asynchronous (n = 21)	%Course or more* (n = 28)
*Course delivery method*				
The course objectives were clearly stated or presented	18 (71.7)	18 (91.4)	18 (86.8)	23 (83.2)
The content was relevant to my needs	16 (62.3)	17 (84.5)	16 (76.3)	21 (74.5)
The course objectives were fulfilled	17 (67.9)	18 (89.7)	17 (84.2)	22 (80.5)
I would take another course via this delivery method	19 (77.4)	18 (89.7)	20 (97.4)	24 (87.3)
The allocated time for the course was appropriate	16 (62.2)	16 (82.8)	19 (89.5)	21 (77.1)
The pace of the course was appropriate	17 (66.0)	16 (79.3)	19 (89.5)	21 (77.2)
*Course content*				
The course content was organized and easy to follow	17 (66.1)	18 (89.7)	17 (84.2)	22 (79.8)
My knowledge and competence in this subject matter increased	18 (73.6)	18 (89.7)	19 (89.5)	23 (83.9)
I have gained useful competencies from this course	17 (69.8)	17 (84.5)	18 (86.8)	22 (79.9)
I am satisfied with the overall quality of the course	17 (66.1)	17 (86.2)	17 (84.2)	22 (78.5)
I would recommend this course to others	18 (73.6)	16 (82.7)	17 (84.2)	22 (79.8)
I am willing to participate in future courses held by GHI	20 (81.1)	19 (98.3)	20 (94.7)	26 (91.3)
*Instructors*				
The instructor was well prepared and knowledgeable about the topic	19 (77.4)	18 (91.3)	19 (92.1)	24 (86.6)
The instructor communicated information clearly and effectively	19 (75.5)	17 (86.2)	18 (86.8)	23 (82.5)
The instructor stimulated interest in this subject	16 (66)	16 (82.8)	17 (84.2)	21 (77.1)
I am satisfied with the overall performance of the instructor	19 (77.3)	17 (86.3)	18 (86.9)	23 (83.3)

^*^Course or more refers to the overall number of participants who completed the entire diploma

##### Course evaluation (open ended responses)

###### Course opinions and expectations

Short-term course evaluations indicate that the course content was positively assessed. Courses were considered organized, comprehensive, and well-explained. Inclusion of practical assignments and case studies was appraised. Courses exposed learners to new concepts and allowed learners to acquire new knowledge and skills pertinent to learners’ job duties and responsibilities. Courses delivered via online learning modality were positively evaluated in terms of course design, layout, flexibility in time management, and the opportunity to facilitate their learning experience through posting discussions on an interactive platform. Overall, the courses met the learners’ expectations.

However, limitations were also mentioned. Learners noted that excessive theoretical information was presented, some material was repeated across several courses and thus became redundant, some courses were too fast-paced, and some assignments along with their instructions were not clear. Additionally, learners indicated that assignments were expected to be more comprehensive and sought for more enriching feedback on assignments and quizzes. Learners also noted that engagement was limited due to lack of direct communication and discussion with the instructor.

###### Suggestions for improvement

Suggested improvements were related to reducing content overload, minimizing content redundancy, and providing more time to cover the content. Suggestions to incorporate interactive material, more visuals such as videos and audio recordings as well as more practical examples and case studies was noted. Some learners called for revision and clarification of assignments to make them more representative of the courses, and to shorten quizzes and modify certain question items.

### Long-term outcomes

Data from semi-structured interviews was collected from 8 participants who completed the diploma. Four organizational surveys were collected from colleagues of learners, and the results obtained from semi-structured interviews and organizational surveys were triangulated. The results are presented in Table 6, under the following two categories: Strengths, and Barriers and Challenges.

**Table 6:** Presentation of Qualitative Data and Emerging Themes

Category 1: Strengths
**Theme 1: Online learning modality**
Codes:
New, pleasant, and beneficial learning experience that encouraged future participation in online modalities
Learning objectives and expectations of courses were met through the online learning modality
Allowed for better time-management, planning, and organization
Flexibility with timing offered allows the diversification of learners from different organizations and different countries
Reliable and credible information is provided in a time-efficient manner, along with proper guidance and support from instructors
Facilitated accessibility to courses at one’s own convenience
A reference of information that can be referred to any point
Self-paced learning allows for enhancement of learning process and uptake of information
Allowed course content to be comprehensive and inclusive
Online platform design was user friendly, well-organized, and easy to navigate
Variation in presenting information was positively perceived and facilitated comprehension of information
No hindrance of communication with professors as they were available and responsive via email
Convenient for those who are not comfortable in a class setting
Decreased gender-bias and increased acceptance in work setting
**Theme 2: Significance of diploma**
Codes:
The availability of a diploma in the humanitarian field that is very much in demand and interest, was perceived to be unique, and not easily available, especially in the middle east setting
Diploma from a reputable institution, such as AUB, was regarded as a major added value to the CV
**Theme 3: Course content**
Codes:
Course objectives were clearly stated and matched its content
Course was well structured and delivered in a detailed manner. The provision of details allowed learner to respond to workplace needs
Diploma is complementary, beneficial, and covers all the information needed in the humanitarian work setting
Content was considered to be novel despite previously attending several trainings in the humanitarian field
Increased engagement due to originality of content allowed for acquisition of information directly from an expert in the field
Assignments were suitable, fostered greater understanding, and were applicable in the work setting
**Theme 4: Instructors**
Codes:
Instructors were knowledgeable, cooperative, supportive, and met high professional standards
Instructors seemed eager to convey knowledge and personal experience in a simple and effective manner
Enhancement of abilities due to instructor’s superior knowledge and experience in the humanitarian work
Instructors provided constructive feedback in a timely manner
Having a variety of specialized facilitators was positively perceived
**Theme 5: Translation of learning into performance**
Codes:
Significant attainment, enhancement, and clarification of previously established knowledge and skills, particularly those that are applicable in areas of conflict
Enhancement of knowledge and skills translated into improved work performance and expansion of scope work
Development of skills that allowed learner to become an independent learner
Increased understanding of organizational work and coworker’s duties in the humanitarian field allowed for effective engagement and coordination with work peers
Improved self-evaluation through setting indicators to monitor work performance
Learner became more systematic in his/her approach to work, as he/she understands the aspects and processes of humanitarian work better
Increased capacity and output in terms of carrying out online sessions to other learners
Increased initiative to be involved in humanitarian projects and reported positive feedback from learner’s co-workers
Introduction of new methods to carry out work-related tasks; such as material/templates provided by the course
Implementation of policy changes and involvement in strategic planning on the organizational and national level
**Theme 6: Personal development**
Codes:
Increased familiarity with overall concept of the humanitarian field increased capability and readiness to work on humanitarian projects
Enhanced knowledge allowed for career and personal advancement in the humanitarian field
Learner encouraged to complete more diplomas that certify his/her expertise in the humanitarian field and to seek out more educational opportunities
Online learning modality allowed for continuity of education especially in regions of instability or in times of a global pandemic
Learner considers his/her input to be more valuable and considers himself/herself to be an asset
Increased self-confidence as a result of the proper guidance that ensured conveyance of accurate information
Increased perceived self-efficacy in carrying out job-related tasks
**Category 2: Barriers and challenges**
**Theme 1: Barriers to applying changes in behavior and performance**
Codes:
Limited ability to learn new skills due to lack of practice
Inability to change work performance or practice due to time constraints, COVID situation, and restrictions by scope of work
Inability to initiate major changes in policies due to time constraints, employment status, and COVID situation
Inability to implement new strategic plans as this is beyond some of the learners’ capacity, expertise, and scope of work, and due to preexisting functional policies
**Theme 2: Engagement and interaction**
Codes:
Doesn’t allow for proper engagement and interaction with peers and professors
Diploma lacked an important aspect which is the exchange of experience and knowledge with people from different backgrounds
Learning process was hindered due to minimal engagement; especially with courses that are not theoretical and require practice
Doesn’t allow for proper engagement and interaction with peers and professors
**Theme 3: Pedagogical approach**
Codes:
Diploma didn’t sustain same quality level, as standard decreased gradually
Negative feedback on excessive delivery of theoretical rather than practical information
Presence of information that was considered basic and insufficient for those with previous experience
Negative feedback on time allocated for some courses as some could have been presented in a more concise manner, while others needed additional time to be covered
Redundancy and overlap of some information
Interactive blocks included an overload of information
Suspected incoherence between course content and some assignment questions, and across some courses
Lack of clarity on some assignment questions
Concerns over exams’ lengths considering the unstable internet connection and the learners’ personal preoccupations
Administration of quizzes at the end of the course; rather than during, hindered proper self-evaluation
Evaluation through MCQs, rather than written, was considered to be inefficacious
Limited feedback or corrections for some exams and assignments

#### Strengths

Five themes emerged under the strengths category: (1) Online Learning Modality, (2) Significance of Diploma, (3) Course Content, (4) Instructors, (5) Transfer of Learning into Performance, (6) Personal Development.

##### Theme 1: Online learning modality

The online learning modality reportedly allowed for better time management, planning, and organization, providing learners with the added flexibility to manage their work and courses simultaneously. Some respondents expressed that this modality provided a pleasant and beneficial experience. Learners also noted that most learning objectives and expectations from the courses were met through the online modality. Importantly, some learners reported that this was their first time attending an online training and were now encouraged to attend similar engagements in the future.

###### A.F.5

Having the diploma online helped me a lot from all aspects, to be honest I was working in Beirut then I moved to Zahle, and at the beginning I was accessing all my courses while in Zahle, so I was thinking that if this diploma required physical presence, I wouldn’t have had the opportunity of exposure to the diploma, so it was way easier for us since we already had work.

Additionally, most learners reported that the online modality facilitated access to courses at one’s own convenience, providing additional flexibility to learners. It was noted that the ease of access to comprehensive information allowed learners to obtain a library of knowledge that can be referred to at any point. Several respondents highlighted the importance of this modality in enhancing their learning process as it allowed learners to self-pace their learning while giving them enough freedom to delve deeper into the course content. Some respondents also appreciated the fact that this modality allowed the provision of reliable information in a time-efficient manner, supplemented by proper guidance from instructors without compromising quality of training. Another respondent notably commented that the flexibility with timing allowed the inclusion of diverse learners from different organizations and different countries.

###### K.Q.1

I got the eight courses, which are very very useful and essential for any project manager who is in the humanitarian setting; all in one place. You have, it is like you are building your own library for the humanitarian setting; you learn through it, but also you have your own library to go back to. It is a reference whenever needed, but this is an amazing learning process to me.

Moreover, some learners commented on the suitability of the online platform, describing it as user-friendly, well organized, and easy to navigate. Learners also mentioned that the variation in pedagogy, such as through videos and interactive blocks, rendered the courses more engaging and facilitated the comprehension of information.

###### KQ1

The online platform was easy to follow but also the way the layout was really nice and gives you where you really are where you are at, at anytime which is really good.

Another learner indicated that online delivery of the HLD allowed them to simultaneously work and study, despite residing in a conflict-affected setting where such a combination is difficult. The diploma was also considered as an added value in an area of instability.

###### K.H.8

You know the war in Yemen impacted the quality of the education and caused it to be unstable. Also, to be able to study and work at the same time was seen as something very hard, so the online course has become the best thing for employees who work and study at the same time. Even if I was enrolled in community medicine, honestly my education at this point was not stable, so for this reason the online course really benefited me.

##### Theme 2: Significance of diploma

The importance of HLD revolves around its regional significance, local development, online delivery, and being tailored to the humanitarian context in the MENA region. Several learners commented that the availability of a diploma focusing on the humanitarian field as being rare and not easily available in the region. Most learners perceived the diploma to be unique, and very much relevant, timely, and of interest to the global population, especially being implemented by a well-known institute at the regional level.

###### K.Q.1

I rarely very rarely come across this kind of diploma especially say in middle east countries there is no such institutes on the ground that teach this type of teaching like humanitarian settings; you hardly come across any institutes or universities that teach you this. So this kind of diploma is unique by itself it is a strength, and I am sure if many people that knew about it, they all will take it because it is needed.

Several learners expressed that the implementation of the diploma by a reputable and well-known institute was regarded as an added value to their resume.

###### K.Q.1

Having this course coming from a very well-known university which is AUB is a very strong point.

##### Theme 3: Course content

Contents of the diploma were evaluated positively by most learners. Most learners reported that the content was appealing, complementary to their jobs, beneficial, and covered essential information in humanitarian settings. Furthermore, the comprehensive nature of the courses allowed learners to gain a rich learning experience and obtain useful resources.

###### K.Q.1

Full of good materials that were gathered in one area; it really helped me a lot. The whole training gets really complementing each other and I think that’s what a professional project manager a humanitarian project manager will need, so it’s the content really well taught throughout and that's all what a project manager in a humanitarian setting will need.

Additionally, most learners reported that the courses provided basic information that was essential for leadership positions, even for those not working in humanitarian settings. Others mentioned that some of the content was novel to them and thus the HLD exposed them to relevant information pertinent to the humanitarian sector despite having previously attended several trainings in the humanitarian field.

###### S.K.2

We need to have all the basic information from the course subjects, and we were able to reach this point.

Moreover, most interviewed learners indicated that the course content was well-prepared, well-structured, and thoroughly explained. Most learners also reported that the course objectives were clearly stated and matched course contents. This enhanced learners’ comprehension of major aspects of humanitarian settings as well as improved their ability to respond to their job requirements. Two learners specifically noted that the inclusion of interactive features into the course content like interactive blocks including images, videos, flashcards, and multimedia blocks rendered the course content to be sufficiently detailed and increased learners’ engagement.

###### N.B.6

Honestly, I found the lectures posted online to be very informative and very interactive with the inclusion of videos and examples … Those were very engaging.

Some learners also appreciated the availability of supplementary material like PDF links and book references. The presence of these resources enriched their learning experience, especially since they were perceived to be credible sources.

###### K.H.8

There were subjects that I wanted to learn more about which advocated for me to read more about them, especially that I received PDF links, so I tried to go over those links, read the shared PDF books and followed up with the courses.

One learner highlighted that the course content seemed to have been informed from the instructors’ personal experience and expertise and did not include any copied or plagiarized material. The originality of the content presented, along with the information directly obtained from experts in the field accentuated the diploma’s distinctiveness and increased the learner’s engagement.

###### N.B.6

Like what I really enjoyed about the course was despite the presence of a reference for it, the lectures themselves didn’t make me feel like I was studying from a book, it made me feel like I was studying from a person who actually knew in the field.

Additional importance was also attributed to the assignments. Many learners described the assignments as suitable, practical, and fostering greater understanding of course material.

###### A.F.5

A strong point of the exercises at the beginning is that we were working on case study proposals. We used to work with case study proposals and use them as examples. This was a strong point because we were really practicing everything that we were learning.

Several learners also commented on the practical use of the assignments in their workplace roles and responsibilities. Learners specifically mentioned that the assignments enhanced their practical experience, accentuated their discussions and interactions with colleagues, and improved their understanding of colleagues’ roles within their organization.

###### A.W.4

I tried to use the assignment in my project with the modules that I was undertaking in order to present both the assignments for the training program and at the same time to present them for my proposal at work. So while doing the assignments, I was at the same time using them in my project in order to incorporate improvement plans, since I am using the same information from the training.

One learner also highlighted how the inclusion of quizzes at the end of each course module allowed learners to self-evaluate their learning and comprehension level.

###### KH8

There was a quiz in the verbal non-verbal communication course. I really liked this idea because it allowed me to be assured that I mentioned everything and that I understood the main idea; it also showed whether I understood the questions that I had to answer. The quizzes served as small evaluation tools for us to know what we understood and what we didn’t. The quizzes also allowed for revision of the correct and incorrect answers; so this was really beneficial.

##### Theme 4: Instructors

Overall, it seems that the performance of instructors in this diploma was well received. Instructors were described as highly knowledgeable and cooperative. They reportedly met high professional standards and conveyed knowledge in a simple and effective manner. One learner reported enhancement of their own abilities specifically due to the instructor's elaborate knowledge and expertise in humanitarian work. An appreciation was expressed for instructors who showed an effort to include personal experiences. According to learners, the inclusion of personal experience rendered the courses more engaging. Instructors also provided constructive feedback in a timely manner and were noted to be ready and available to answer all questions via email. In addition, having a variety of specialized instructors was considered one of the strengths of the diploma.

###### K.H.8

I also liked the idea of receiving feedback from instructors whenever we sent them the assigned tasks. I liked that they provided grading and feedback and what should the answer for each question be.

##### Theme 5: Transfer of learning into performance

Most learners reported acquiring and/or enhancing their knowledge and skills through the HLD. The information provided through the courses was reportedly beneficial in complementing previously acquired knowledge and provided appropriate guidance for carrying out humanitarian work, particularly in areas of conflict.

###### A.J.3

In the M&E course, I used to work on basic reporting which is to report my activities, but now I am doing reports for the whole team. I know that M&E is a cornerstone, and this I was able to acquire from the course, and I learned also how it plays an important role if you want to do a proposal, let's say for a project, so you need to have your budget drafted and your M&E log frame drafted.

Learners reportedly gained new skills that influenced their practice, such as team management skills, budgeting, negotiation, and communication skills, all of which allowed them to expand their scope of work, increase their involvement in tasks, and improve their overall performance.

###### M.Z.7

Adding the management aspect to the things helped a lot; it helped me to understand more what is happening in the project. Previously for example I was involved in writing proposals but I was involved in the technical parts only. I didn’t go, for example, in the monitoring plan or in, let’s say budgeting, and these two things were part of the diploma, and after that I started to be more involved; I started to suggest things and see things, and this proves valuable with my technical background.

In addition to reporting that the training familiarized them with the roles of their coworkers, learners noted that this training allowed them to have a holistic understanding of organizational structures and processes in the humanitarian field. This, in addition to the acquired knowledge, allowed some of them to engage and coordinate more effectively with coworkers. For instance, one learner reported an improvement in internal coordination with his coworkers after gaining a better understanding of the process of logistics implementation. Other learners also reported implementing new skills such as self-evaluation through setting indicators to monitor work performance, becoming independent learners, and increasing their capacity and output to deliver online sessions to other learners.

###### A.W.4

I am now more goal oriented and I am able to put more indicators so I can trace my performance or the activity that I’m doing, so from the start I can think in a more strategic and systematic manner instead of being somewhat random so when I’m doing any activity or anything else, I am focused on the end-goal and I can do evaluation routinely and clearly from the start.

Importantly, one female learner reflected on a positive experience following completion of the diploma whereby she encountered decreased gender-bias and increased acceptance in her work setting. The diploma had enhanced her skills which translated into being better accepted in a gender-biased work setting.

###### K.H.8

Honestly, I benefited from the diploma in my workplace. I believe that the male’s thinking that he remains better in a managerial position in comparison to a competent female manager has changed. The acceptance of me as a competent manager and a knowledgeable woman rich with information has increased.

Although this was not commonly reported, the training did allow few learners, mainly coordinators and those in managerial positions, to develop new policies and to participate in the strategic planning of their organization. One learner reported that the training enabled him to co-establish a humanitarian non-governmental organization.

###### K.Q.1

I re-established a new organization as an international organization, so I am one of the co-founders, and its headquarter is in Germany. But I believe this course enabled me to be able and to be in a position that I could establish this organization; it is a humanitarian non-governmental organization.

Many of these findings were also corroborated by data collected from learners’ colleagues through the organizational surveys, for instance one supervisor reported that *“He has improved in establishing relevant indicators and knows exactly where and when to be used, that has impacted well the improvement of his department general performance”*. Another stated that* “A.W.4 has prepared a proposal for a quality improvement plan but the lack of required authority would be the biggest barrier to implement his innovative thoughts”.*

##### Theme 6: Personal development

Many learners highlighted the role of HLD in furthering their personal and professional development. Some learners reported increased self-confidence and others noted improved capability and increased readiness to work on humanitarian projects following the courses.

###### K.Q.1

This knowledge I gained and the experiences through the courses made me more confident that I am more familiar with humanitarian work now.

Several learners also reported that their acquired knowledge allowed for career advancement in the humanitarian field. One learner also noted the significance of the diploma in his personal life, particularly because a degree in humanitarian work is well regarded given the extensive humanitarian presence in his country.

###### A.J.3

My knowledge increased, and this helped me evolve in my work, because I started as a PSS officer and then became the senior, and the thing that helped me most was the fact that I learned at Global Health Institute how things should go, so I paved my way with the help of this program.

One learner emphasized that the diploma encouraged him to complete further certifications in the field of humanitarian affairs. Another learner explained that the diploma allowed him to acquire in-depth knowledge of a field he was unable to learn about previously due to it being slightly different from his original academic specialty.

###### M.Z.7

Okay so the training exposed me to these topics that I wasn’t aware of before and it showed me the importance of having the leadership aspect, the management aspect in my work and based on the… like what we learned in the diploma was the basics, I decided to go further into that and proceed with an MBA.

#### Barriers and challenges

Three themes emerged under the category of weaknesses and challenges: (1) Barriers to Applying Change in Behavior and Performance, (2) Engagement and Interaction, (3) Pedagogical Approach.

##### Theme 1: Barriers to applying changes in behavior and performance

The most notable challenge faced by learners who were interviewed was associated with translating the acquired information into organizational changes. This was especially true for learners who are not coordinators or do not hold senior positions. Some noted that they were unable to apply their newly learned skills within their work due to lack of practice, being newly employed, or contextual factors. Others attributed the absence of knowledge transfer to time constraints or to the impact of COVID-19 precautions on restricting the application of various work-related duties during this period. Few participants noted challenges in initiating major changes in organizational policies or in implementing new strategic plans. Learners commented that such changes could not be carried out at the individual level alone; rather, they require structured changes with the involvement of coworkers at different levels of the organization. One respondent reported that the preexisting functional policies in their organizations do not allow for the implementation of new ones.

###### K.Q.1

I was with another organization, but they phased out from the country since June, had two months sitting and resting, then I joined another organization and you know this pandemic came in and it did not allow, it did not allow to fully apply the knowledge I gained on the programs I was doing.

##### Theme 2: Engagement and interaction

Despite the advantages offered by an online learning modality, one limitation reported by most learners who were interviewed was the minimal engagement and interaction. Specifically, asynchronous courses were perceived to be less interesting than others.

###### A.J.3

At certain times you are not engaged, there is no engagement. This lack of engagement will make you bored. So, if you have this course, it is a dry material. If we are also accessing two or three chapters each week, we are just reading them, if there is no interaction it is going to be very dry.

It was additionally reported that the online learning modality limited the exchange of experience and information between people from different backgrounds. As such, learners noted low levels of interaction between learners. Some respondents expressed the need to have at least one session in which all learners are present, as this would enhance not only participation but also the sharing of expertise.

###### M.Z.7

It just needed more encouraging the exchange between the participants more because usually when I attend any session by when I attend any training session, any workshop, one of the most important parts for me personally is the exchange between the participants because participants have different backgrounds. They have different experiences; they would share lessons learned and best practices with each other, and maybe this was what was a bit lacking in the diploma.

Moreover, learners mentioned that the limited ability to interact with instructors and ask questions directly hindered the clarification of some course content and obstructed the learning process. One learner suggested interacting with instructors through posting questions and answers on the modules or through weekly live sessions as it would have added value to the training. It was also noted that the learning process was hindered by minimal engagement, especially for courses that deliver practical content rather than theoretical.

###### A.J.3

For example, for the personal communication skills, this is a skill to practice you have to exercise, use your verbal and non-verbal skills, this thing was being applied, you have to use eye contact language and all this stuff; you only know them theoretically, you get me, and same applies for negotiation, okay you can write…but the engagement is lacking.

##### Theme 3: Pedagogical approach

Although the course content was well received by most learners, a few shortcomings were indicated. Several learners mentioned that the diploma did not sustain the same quality throughout its implementation, noting that the quality seemed to gradually decrease towards the end of the diploma. This was due to an increased focus on presenting theoretical content, without enough provision of practical assignments and exercises.

###### A.F.5

The first three four courses were very interesting. A high involvement from the participants was observed; we had a lot of projects to work on, but the involvement decreased throughout the year. In my opinion, the level of the diploma deteriorated a lot, and it didn’t maintain its initial level of quality. With each course, the quality level of the diploma was declining, specifically our level of participation and involvement with other attendees. Knowledge wise, the diploma became solely theoretical at the end. We almost stopped doing course homework and exercises. So, by the end of the courses, we didn’t feel ourselves really benefiting from the course by the end of the courses. The modality of the exam, the course became more theoretical, very very theoretical.

Another concern was reported by a learner who expressed feeling overwhelmed by the volume of material presented behind the interactive blocks as it affected the learner's attention span. Another learner highlighted the presence of repetitive and overlapping information across the courses which negatively impacted the learner’s enthusiasm to proceed with the studying process.

###### N.B.6

If you are taking six to seven courses and for a whole week you are learning the same information, you will get bored if you read for six to seven weeks the same information; maybe changing the flow of the course, given that the courses are sequential one after the other, there should be a better way for the teacher to communicate the content and avoid the overlapping of the information. I felt there was a lot of overlapping information, and this made me feel mad at times because I had to read the same information. Given that we are encompassed with the course for a whole one year and a half, the overlap of information I think is the most important thing that you need to take into consideration because it really gets repetitive.

Several learners perceived the content of the diploma to be insufficient for those with a vast background in the humanitarian field. Conversely, some learners reported that there was minimal accommodation within the course assignments to those with limited humanitarian background, stating that assignments seemed to target individuals who were already employed at an NGO.

###### S.K.2

The diploma was addressing individuals from different backgrounds, so it was not hard for us to follow-up with the content. However, the assignments implemented across the courses asked us to provide examples based on the NGO that we were working at; like for instance in the Finance course, we had to provide examples of budgeting numbers, and I, however, was not subordinated to any NGO, so it was hard for me to complete the assignment. Not all of us are affiliated with an NGO and some of us were taking the course just to be able to work in an NGO sometime in the future.

Few learners provided negative feedback for the time allocated for course delivery. Several learners reported that the videos were quite long and could have been more direct and concise while others required additional time to cover the material adequately.

###### K.Q.1

Yeah, the content as a whole of 8 courses is sufficient and I think it is very useful, but shortening some videos, shortening some materials; I kind of felt some of the facilitators they put all the materials they came across that is related to the subject that is not necessary need to be there.

One learner indicated that presenting the course content over a longer duration would have given them more time to manage and plan for the course. This same learner also suggested for the diploma to be delivered by expert professionals who can teach the course in the Arabic language.

###### K.Q.1

The content of the course was not appearing in the platform on say enough time for the student to plan; it is like my studies tomorrow and the course content could have appeared today which may be ruining my other plans especially it is online course so usually most of the people are working either full-time or part-time. So, these are to say maybe weaknesses.

Several concerns were reported regarding the assignments and quizzes. One learner noted that towards the end of the diploma, less focus and importance was directed towards assignments, quizzes, and grades. Another concern highlighted the lack of feedback by instructors on assignments and quizzes.

###### K.H.8

When the instructors used to administer quizzes for us, we would send them back the quizzes. However, we would not know if our answers were right or wrong. We didn’t receive any reviews. We would study but by the end of the day not know if our responses were correct or wrong. So how would I learn? Shall I learn only from reading the course?

Some learners were also concerned with the change in format of assignments and quizzes from written responses to multiple choice questions. A preference for written rather than multiple choice questions was noted as they allowed learners to solidify their newly acquired knowledge.

###### N.B.6

I think one of the courses that I enjoyed the most was the negotiation in the hostage prices course mainly because the assignments weren’t multiple choice questions. The teacher actually asked us to write a paragraph and try to explain something according to a given format or framework. So, I believe these types of assignments are way better than multiple choice questions. I think there could have been a better way to evaluate the student rather than just using multiple choice questions.

One learner noted inconsistencies between the course content and some assignment questions indicating that the knowledge assessment and evaluation quizzes were not representative of the course lectures and material. They recommended improving the course evaluation and knowledge assessment measures. Another learner mentioned that quiz questions were incoherent, and objectives were not clearly communicated.

###### N.B.6

The quizzes in my opinion were not really representative of the lectures given that there was a lot of knowledge in the courses, and there were like 10 questions among which 6 were representative of the content, but the other 4 questions were not representative over the content. So, I would say maybe if you try to improve the evaluation of the students for the course that would probably be the biggest thing to improve on, and I perceived this to be the biggest weakness.

One learner additionally reported that the administration of quizzes at the end of the course, rather than during, hindered adequate self-evaluation. The same learner provided negative feedback on the length of the quizzes and assignment.

###### K.H.8

The instructors were administering tests at the end of each module, so the information was accumulating by the end, and I was not able to be assured throughout the courses whether I really understood the material or not. Another thing that I disliked about the course is the big number of the questions; there were courses when the number of the questions reached to 40–50, so it was really hectic.

## Discussion

Humanitarian crises and emergencies fundamentally characterize many of the MENA region’s countries, and they have been escalating in magnitude and severity over the past decade. Although many of them are a result of natural disasters, the vast majority are triggered by social, political, and security turmoil along with armed conflicts that have devastating effects on affected countries and populations. One such example is the shift in defining conflicts as becoming protracted and lasting for long periods of time, as can be seen from the wars in currently occupied Palestine and Syria [29, 40, 41]. These conflicts have triggered massive displacement of populations and have had a significant impact on many countries worldwide, especially on neighboring countries.

Despite that, along with the obvious need of affected countries within the MENA region to be able to respond to such conflicts across various sectors, many challenges have been reported. Perhaps the most significant of those challenges is the dependence on international actors to lead the humanitarian response, the brain drain of local professionals, and the limited opportunities for local actors to develop the needed knowledge and skills to operate in such a complex climate. [29].

In light of these serious problems, the Global Health Institute at the American University of Beirut developed the Humanitarian Leadership Diploma to trigger an important shift and to fill a neglected gap in the response of humanitarian actors within the MENA region. Capitalizing on its regional leadership position within academic research and education, and through HLD, GHI aimed to provide one of the very few credible, comprehensive, and locally developed and contextualized training programs to build the capacity of local actors and supply them with the necessary knowledge and skills needed to work in the humanitarian field. To enhance accessibility for learners from across the MENA region, the program was designed to be fully online.

The need to build local capacity and to decrease reliance on international actors is well established in the literature, however there are very limited local opportunities available to prepare, train, and educate humanitarian workers using contextualized knowledge. This is especially true for online learning, whereby to the best of our knowledge, no studies have been published to-date evaluating such a training program from the MENA region yet. The need for innovative capacity building initiatives, and especially evaluation of online learning programs within the MENA region has been reported to be a necessity within the literature [42, 43]. With the advent of the COVID-19 pandemic that prompted major shifts to online modalities [44], and in combination with the increasingly complex humanitarian crises affecting the MENA region, such an evaluation is crucial to better understand the individual and organizational-level impact of such programs, and to subsequently guide future efforts. In the present study, we report the first evaluation of the HLD program.

In general, the overall project was considered to be successful because of multiple factors such as its timeliness and relevance to current circumstances, accessibility and strong coverage across countries in the MENA region, its design, course content, and delivery method, low dropout rates, and the short-term and long-term positive impact reported by most learners, to name a few. That said, many shortcomings were also reported such as learner engagement and consistency of course delivery, along with others such as pedagogical approaches, presentation of material, and the limited opportunities for interaction between peers and instructors.

One of the most commended aspects of the diploma was its complete reliance on online learning. This was reported to be a key strength for different reasons. Professionals working in this field experienced significant advantages to traditional learning such as having enough flexibility to plan for their work-study schedule and to better manage when and where they attend the courses as it fits them. Importantly, because this course aimed to target humanitarian actors across the MENA region, this would not have been possible to do had it been delivered in-person because of challenges traveling across countries [45, 46]; considering the lockdowns and movement restrictions imposed during the COVID-19 pandemic, travel would have also been difficult within-country. With this flexibility, participants reported being able to better self-pace and to have the ability to stop and resume lessons as needed. It is also necessary to recognize that this program, by virtue of its online delivery, provided key learning opportunities for key populations who would have not had access to this material otherwise during this period.

The short-term quantitative data indicated that learners’ knowledge significantly improved in all courses and their satisfaction was generally high across all courses; this means that the online diploma does have merit as an educational tool for the participating humanitarian workers in the MENA region. From the interviewed participants, the diploma’s content was reported to be comprehensive, to complement the fields that they worked in, and to provide them with knowledge that aligned well with their roles. This is an important finding because the diploma was designed to cover the essentials of humanitarian leadership, to consolidate fragmented knowledge of participants, and to provide them with a solid foundation in humanitarian knowledge and skills, knowing that these topics are rarely covered in formal educational settings [47, 48]. Indeed, there is widespread consensus among the humanitarian community of a rise in the demand for professional training in humanitarian action that is not satisfied via traditional training methods [47]. Consequently, there has been a proliferation of online courses and certification programs offered by organizations to ensure adequate training of humanitarian workers [47]. However, such programs or certifications are rarely evaluated for efficacy, perhaps due to the unique demands of humanitarian training, the challenging nature of conflict settings, and the necessity to adapt conventional monitoring and evaluation strategies for use in conflict settings [49]. For example, humanitarian professionals often work in underprivileged areas and remote regions where access to resources and travel may be limited due to sociopolitical turmoil and insecurity. Additionally, humanitarian workers operate across various sectors, and meeting and evaluating their capacity building needs may depend on the size, resource base, sources of funding, and technical capacity of their organizations, all of which vary based on location and context of operation [47, 50]. That said, participants appreciated the fact that the courses within the HLD were developed and delivered by professionals in the field who are locals from the MENA region, mainly because of their contextualized knowledge and experience to which they could relate. Additionally, it was important for participants to recognize that the platform provided a depository or a library of information that they could access on-demand and thus provide them access to global material that was locally tailored.

Despite these strengths, it was reported that the online modality presented some challenges, mainly in relation to the level of engagement with instructors and peers. Understandably so, the engagement experienced during in-person classes may be difficult to replicate in online settings where learners’ presence and interactions may be forced and thus neither beneficial nor motivational [44]. Surely, online learning forces educators to rethink instructional techniques and the relevance of ‘seat time’ as a standard measure of educational credits [44, 51]. As such, more consideration should have been placed regarding this aspect when designing and delivering the course. For example, this could have included access to forums where participants can meet with and engage with each other, or organizing a weekly schedule whereby calls can be made with course instructors to ask questions or to share comments. Learner engagement is key in such programs and more emphasis should be placed on optimizing it in challenging settings, such as in e-learning. Although initially designed to be an online synchronous program to ensure interactions between learners and instructors, coordinators of the program had to re-shift the modality midway to include an asynchronous approach. This is because the former presented difficulties in matching learners’ schedules, accounting for time differences, and overcoming internet connectivity issues, which is why learners and coordinators opted for a mix of synchronous and asynchronous approaches [52–54].

Some of the main challenges related to the reliance on a theoretical pedagogical approach when delivering the courses, that in many cases lacked enough interactions between instructors and peers. Although participants could reach out to instructors should the need arise, many reported preferences for integrating more practical approaches in the delivery of courses to enhance engagement and reduce boredom. This was especially true in the case of receiving feedback regarding their assignments and quizzes, whereby minimal feedback was relayed back to them, and this was reported to be problematic for many. Rightfully so, active learning is key to improving the learning experience whereby learners adopt an active role in the process as opposed to being passive receivers of information [55, 56], and this should be considered when designing and delivering such programs online. Additionally, participants reported preferences for receiving concise key information rather than being overwhelmed by large volumes of material. Such challenges are echoed by other capacity building programs aimed at health workers in fragile settings [57], suggesting the need to prioritize practical and engaging approaches that deliver a suitable volume of information in future capacity building programs.

Another element that yielded mixed findings is the transfer of knowledge into organizational practice. Some participants reported direct effects such as increases in their ability to perform their jobs, to improve organizational policies and strategic plans, their better manage their work, and reported having a better understanding of how humanitarian organizations work and what the roles of different departments are; this was corroborated by some organizational-survey responses completed by learners’ peers and supervisors. Although for some there was some translation of knowledge into organizational practice, for others this was not the case due to different reasons such as them not occupying roles that are senior enough—which meant that some of the learnings could not be directly applied given their job descriptions—or due to specific organizational policies. Nevertheless, the knowledge gained remains important to them and may indirectly influence their organizational practice and humanitarian knowledge.

For instance, despite the fact that not all participants were able to directly apply the skills and knowledge they gained, many highlighted the fact that obtaining a certificate in humanitarian leadership from a credible institution in the region was regarded as an added value to their resumes. This is because of the dearth of similar and accessible certificates, and the increasing need for qualified humanitarian workers among humanitarian actors in the MENA region. In this regard, many participants reported increases in their confidence and ability to improve their personal and career development.

## Limitations

Our study generally suggests that such programs are feasible and effective and should be scaled to account for more topics reaching wider populations. This, however, should be interpreted in light of important limitations, such as our inability to collect long-term data from a larger sample due to limited responsiveness of some learners, and the fact that many did not complete the full diploma. Moreover, only a handful of learners’ peers were willing to complete the organizational-level survey, which hindered our ability to comprehensively assess the transfer of knowledge to organizational practice. That said, we did reach data saturation when conducting interviews with learners, indicating that the sample size was adequate. In future implementations, better strategies for data collection, especially at the organizational level should be considered to better assess the larger impact of the program. This may also include more detailed assessments at the organizational level that potentially transcend self-reports of learners and their peers. Finally, important limitations at coordination-level prevented the program from fully materializing as planned, such as drop-out rates, shifting towards asynchronous approaches midway, and general challenges associated with participant engagement and communication. This may also suggest that process evaluations should be integrated in evaluations of similar programs to better capture implementation challenges.

## Conclusion

This is one of the very few studies reporting an evaluation of an online capacity building initiative providing a comprehensive program for humanitarian workers in the MENA region. The value of the program lies in that it is locally developed and delivered, providing solid grounds for scalability in the future. The main strength of this study is its reliance on short-term and long-term individual and organizational level data to assess the reactions, knowledge, and practice improvements of participants using multiple sources. The findings of this study, both in terms of reported strengths and challenges, are critical to informing researchers and humanitarian actors looking to design and deliver online capacity building programs for humanitarian actors in the MENA region. Online learning is now emerging as a go-to modality worldwide, especially following the COVID-19 pandemic, and this is especially important in conflict settings where access to quality education is limited, especially in the humanitarian realm.

Despite it being based on a case study for one program evaluated at the level of outcomes, our findings funnel into key recommendations as delineated below and suggest that:

1. A mix of synchronous and asynchronous approaches are favorable to allow enough flexibility for learners who may have difficulty attending all sessions at a specific time such as for those who balance between working and studying, or who may have connectivity issues, among others.
2. For asynchronous approaches, it is necessary to consider engagement strategies with learners such as providing opportunities for at least one session per week where learners can interact with each other and their instructors in real-time, similar to office hours within university settings, or through designing an online forum where questions and answers are posted in relation to the courses.
3. Design course materials (videos, presentations, and so on) to be concise and to summarize key learnings, while providing the opportunity for further detailed optional readings for learners who wish to delve deeper into specific subject matters. As for exams and assignments, it is important to provide adequate feedback to learners capitalizing on their specific case studies and not only rely on multiple choice questions.
4. Limit theoretical pedagogical approaches during course delivery and provide more opportunities for practical approaches that emphasize active learning. Examples could include case studies of real-time pertinent problems, simulated scenarios with actors, and organizational management such as for budget allocation, task delegation, among others.
5. Ensure that course topics and content are pertinent to current issues, contextualized to the specific region, and that they are developed by professionals in the field who can draw on their experience and expertise when delivering the courses.
6. Develop an engagement strategy to maintain regular communication with learners for the duration of the program, but also to ensure participation in data collection activities in the long term.
7. Given the apparent success of the program largely due to capitalization on partnership between GHI as an academic institution and HLA as a humanitarian actor, consistent emphasis on establishing and maintaining equitable partnerships between such organizations should be considered in future implementations.

## Data Availability

The datasets used and/or analysed during the current study are available from the corresponding author on reasonable request.

## Appendix: Testimonies from the HLD participants

### Participant 1

Thrilled to conclude the Humanitarian Leadership Diploma delivered by AUB Global Health Institute. Having looked through the website of the Global Health Institute at AUB, I was very delighted to find the opportunity to spend one year on learning humanitarian management at the Middle Eastern Center for Humanitarian Advancement. I have decided to apply for this training because I am sure it would strongly help me in my career as a humanitarian activist and a future humanitarian manager.

Effectively, it was an outstanding experience as I got to enhance various skills to address humanitarian disasters. During this course, I learned about effectively managing humanitarian projects and resources, monitoring and evaluation systems, fundamentals of humanitarian leadership and strategic plans disaster response, as well about International Humanitarian Law and International Humanitarian Rights Law in conflict settings.

I’m deeply grateful to the Global Health Institute for granting me this opportunity and to those who helped me throughout this DiIploma! Finally, I feel blessed for the amazing people I have met during this training!

### Participant 2

I've started with Humanitarian works since 2014 after I was forcibly displaced from my hometown and I am a displaced since then.

I started freshly with NGO/Humanitarian works with very less experience and background. I always loved to work and help the people in need during disasters. I was newly appointed Project Manager when I was looking for best fit courses to develop myself in this field. I cam across HLD from GHI/Kaya that I was certain it was the best way to build my capacity, increase my knowledge, and boost my experience. Before taking the course (HLD), I was referencing my work to different resources mostly from internet searches. Doing that was taking big amount of my time and sometimes not fully applicable to my work. Taking the course/Diploma with GHI made me confident on my decisions and references. I used my study of the course as library to go back to it whenever needed. With the course, I was able to make very good improved steps in the iNGO I was working with in terms of assessment ways and forms. With that, I could help other office through sharing my knowledge with other relevant technical sectors and enhance the work.

The learning modules were very informative and comprehensive. I think the courses/modules could have been more beneficial if they would have been equally weighted (about the same amount of information in all the different 8 courses). Also, if there would have been some group or one to one short calls/meetings between the facilitator and the participants. Videos and meetings included in some of the courses were very long and could have been summarized more with bringing in more expertise persons in.

Sometimes the contents of the course were delivered to the platform very late that made the student left with very tight schedule therefore lack of planning for the study. Overall, I benefited from the courses and enhances, boosted, and increased my knowledge in the field of humanitarian. I thank you all for the great opportunity and looking forward for future Min/Master ones. Kind regards.

### Participant 3

I have chosen the HLD offered by AUB’s GHI given its reputation in the humanitarian field in Lebanon and AUB’s in general. I have been exposed to GHI through various events that I attended prior to my enrollment in said diploma and I can say that I developed great appreciation all of the work GHI is doing to improve health for all residents of Lebanon.

The HLD complemented my experience in project management (Health) and provided me with academic insight on leadership in the Humanitarian setting. The introductory course (PM in Humanitarian settings) does an excellent job in providing the needed background for anyone looking for a career in humanitarian work. The practical assignments requested for some of the courses (Budgeting/Monitoring and Evaluation) I consider were spot-on and reflect what to expect from any leadership position in the humanitarian health programs. Other courses were a bit too theoretical for someone of my background (mediation/negotiation skills & Interpersonal communication). I mean I would have preferred more practical assignment engaging other colleagues to learn from each other experience. The interviews with leaders in humanitarian organization were quite insightful though stretched for long periods of time (If my memory serves me well, I recall 1.5 h or more at times). That said, it would be great if GHI can edit such long interview to focus on the highlights (maybe provide long and short versions of each interview).

I have built on the knowledge I gained through this diploma, especially when it comes to the “refreshers” I have received on humanitarian principles, sphere standards, and core humanitarian standards (CHS) to effectively navigate through some tough decision I had to make on ensuring high quality humanitarian programs for the best outcomes of our beneficiaries. I found myself at times clearly referencing specific CHS when taking decisions in adapting program design or revising standard operating procedures. Having that clarity in perceiving situations on the ground and reacting in-line with the above principles/standards have made all the difference to my practice.

## Abbreviations

MENA: Middle East and North Africa
HLD: Humanitarian Leadership Diploma
NGO: Non-governmental Organization
UN: United Nations
ALNAP: Active learning network for accountability and performance
SOHS: State of the humanitarian system
GHI: Global Health Institute
GHLAD: Global Health Learning and Development
HLA: Humanitarian Leadership Academy
SME: Subject matter experts
COVID-19: Coronavirus-19
